# Nicardipine Loaded Solid Phospholipid Extrudates for the Prevention of Cerebral Vasospasms: In Vitro Characterization

**DOI:** 10.3390/pharmaceutics12090817

**Published:** 2020-08-28

**Authors:** Christin Zlomke, Johannes Albrecht, Karsten Mäder

**Affiliations:** Institute of Pharmacy, Martin Luther University Halle-Wittenberg, Kurt-Mothes-Str. 3, 06120 Halle (Saale), Germany; christin.zlomke@pharmazie.uni-halle.de (C.Z.); johannes.albrecht@pharmazie.uni-halle.de (J.A.)

**Keywords:** phospholipid, phosphatidylcholine, lecithin, controlled release, implant, extrusion, parenteral, biodegradable, nicardipine, dihydropyridine

## Abstract

The aim of the study was to develop nicardipine loaded phospholipid extrudates as an alternative for PLA/PLGA-based implants for the prevention of cerebral vasospasms. Extrudates of different mixtures of saturated and unsaturated phosphatidylcholine (PC) were produced and characterized by DSC, microscopy and texture analysis. Single phospholipid components were identified by ELSD-HPLC. Extrudates of 2 mm diameter were obtained by twin screw extrusion temperatures below 50 °C. The ratio of unsaturated and saturated phosphatidylcholine components determines the physicochemical properties of the extrudates as well as the rate of erosion. Nicardipine loaded phospholipids extrudates released the drug over several weeks in vitro. The phospholipid composition of the remaining extrudate changed during the release, the content of unsaturated phospholipids decreased faster compared to the saturated ones. In conclusion, solid phospholipid extrudates are promising materials for the development of new parenteral controlled release systems.

## 1. Introduction

Parenteral controlled release has a high potential to improve drug therapy in different disease areas, e.g., for the release of anticancer drugs, anti-inflammatory agents, antibacterials and antifungal drugs and cell protective agents. Local controlled release might provide much higher concentration in the desired area (e.g., in the eyes, ear [1], brain [2], joints, periodontal pocket) and decrease the overall dose and the side effects in other tissues. It is also clinically used for long-lasting delivery of systemic acting drugs (e.g., peptide hormones for the treatment of cancer or antipsychotic drugs), with the advantage of decreased application frequency, the avoidance of infusions and a higher patient compliance. Both non-degradable and biodegradable systems are used. The advantage of biodegradable depot systems is the avoidance of the removal process of the implant. Currently, the dominating biodegradable materials are polylactide (PLA) and poly-(lactide-co-glycolide) (PLGA). Despite the fact, that the clinically used systems are marketed since several decades, there is a need to improve and to enlarge our drug delivery options, because PLA and PLGA have, in addition to their beneficial properties, also limitations. Their physicochemical properties are quite similar. In addition, polymer degradation leads to the formation of lactic and glycolic acid which are highly acidic due to the alpha hydroxy group. As a result, an acidic microenvironment with very low pH values might develop [3,4], and nonlinear and complex release profiles might result [5]. In our attempts to develop formulations for the prevention of cerebral vasospasms after subarachnoid hemorrhage or surgical clipping of aneurysm, we observed that lower drug loading led to higher amounts of the released drugs, not only in percentage, but also in total mass [6]. Other groups reported on drug PLA/PLGA induced drug degradation, e.g., a covalent linking between PLGA degradation products and incorporated drugs [7]. With respect to the large number of drug molecules with different properties and demands, it would be desirable to have a broader selection of biodegradable materials with different hydrophilicities and release rates. Furthermore, highly acidic degradation products should be avoided. Research efforts include the development of alternative polymeric materials [8,9] or lipid based implants and microparticles [10,11]. In addition to the systems mentioned above, phospholipids are an attractive option. They have an excellent safety profile and are widely used for the formation of liposomal drug delivery systems and as stabilizers for parenteral lipid emulsions [12]. Different approaches have been developed to achieve long-lasting release with phospholipids. These include highly concentrated phospholipid dispersions (e.g., Depofoam™ technology) [13,14,15] and calcium precipitates (phospholipid cochleates) [16]. Surprisingly, only few efforts have been devoted to the formulation of water-free phospholipid based extrudates [17,18,19]. Phospholipid extrudates might have applications both for oral and parenteral drug delivery. However, more detailed knowledge about the drug delivery mechanisms and the impact of the phospholipid composition is required for a rational based development of the systems. Therefore, it was the one aim of the current study to formulate phospholipid extrudates with different phospholipids and to study the impact of the composition on the physicochemical properties, the phospholipid microenvironment and the water penetration and drug release rate. In addition, with regard to the undesired release profile of nicardipine from PLGA extrudates in our previous study [6], we wanted to develop a nicardipine loaded phospholipid implant for the treatment of vasospasms in subarachnoid hemorrhage with an improved and more constant release profile. It is known, that in most cases, vasospasms occur between days 5 and 10 [2,20,21]. Therefore, we wanted to achieve nicardipine release over this time period. Because vasospasms are unlikely to occur during the first three days, a lag time of release can be tolerated, if it does not exceed 3 days. With respect to the high safety and biocompatibility of phospholipids, we decided to avoid other excipients and to tune the physicochemical properties and the release rate by different ratios of saturated and unsaturated phosphatidylcholine. In order to understand and to control the physicochemical properties, the samples were characterized by DSC, light microscopy, rheology and texture analysis. Drug release and phospholipid composition after manufacture and during release was done by HPLC with an evaporative light scattering detector (ELSD). Using this technology, we aimed to get a deeper insight into the drug release mechanisms and to explore the possible different release kinetics of saturated and unsaturated phospholipids and the impact of hydrolysis.

## 2. Materials and Methods

### 2.1. Phospholipids

Phosphatidylcholine type S100 (soybean lecithin, phosphatidylcholine content ≥94%) and S-PC-3 (soybean lecithin, hydrogenated, phosphatidylcholine content ≥98%) were kindly provided by Lipoid GmbH, Ludwigshafen, Germany.

According to the manufacturer’s specification the fatty acids of the two acyl groups of phosphatidylcholine within the used lecithin types are listed in Table 1.

Phosphatidylcholine standards of 1,2-dilinoleoyl-sn-glycero-3-phosphocholine (18:2–18:2 PC, DLPC), 1-palmitoyl-2-linoleoyl-sn-glycero-3-phosphocholine (16:0–18:2 PC, PLPC), 1-palmitoyl-2-stearoyl-sn-glycero-3-phosphocholine (16:0−18:0 PC, PSPC) and 1,2-dipalmitoyl-sn-glycero-3-phosphocholine (16:0–16:0 PC, DPPC) were purchased from Avanti Polar Lipids (Alabama, US). Phosphatidylcholine standards 1-palmitoyl-2-oleoyl-glycero-3-phosphocholine (16:0–18:1 PC, POPC), 1,2-dioleoyl-sn-glycero-3-phosphocholine (18:1–18:1 PC, DOPC) and 1,2-distearoyl-sn-glycero-3-phosphocholine (18:0–18:0 PC, DSPC) were supplied from Lipoid GmbH (Ludwigshafen, Germany).

### 2.2. Drug and Further Materials

Nicardipine hydrochloride (>98%) was purchased by Alfa Aesar (Karlsruhe, Germany). Nicardipine free base was obtained from nicardipine hydrochloride by dissolving the salt in water and pH adjustment to pH 4.5 with 1 M NaOH. The formed nicardipine free base was extracted with methylene chloride. Afterwards the solvent was removed by rotary evaporation and the drug was dried in vacuum.

Buffer salts for preparation of PBS were of analytical grade and supplied from Grüssing (Filsum Germany: NaCl, Na_2_HPO_4_, KH_2_PO_4_) or Fluka Analytical (Seelze, Germany: KCl). The organic solvents methylene chloride, chloroform, acetonitrile, methanol and trimethylamine (TEA) were of HPLC grade and supplied by VWR (Darmstadt, Germany).

### 2.3. Manufacturing of Mixtures

The lipids and if applicable the drug were precisely weighed to the required ratio, transferred to a round flask and dissolved completely in freshly distilled chloroform. After rotary evaporation to dryness the lipid mixtures were further dried in vacuum and stored afterwards at 8 °C with protection from light.

### 2.4. Manufacturing of Implants by Extrusion

To enable implant preparation by extrusion, the lipid mixtures were cryomilled (Cryomill, Retsch Technology, Haan, Germany) in order to obtain a homogeneous dry powder. Approximately 3 g of lipid mixture were ground at −196 °C in a 25 mL grinding jar with 2 stainless steel spheres (diameter 14 mm). After auto-precooling at 5 Hz four milling cycles (1:30 min) at 15 Hz were performed with 30 s intercooling at 5 Hz.

The resulting fine powder was used for extrusion with the twin screw extruder ZE 5 eco (Three-Tec GmbH, Seon, Switzerland) equipped with two double concave screws of 5 mm diameter (L:D 20:1) and a round die (diameter 2 mm). The powder inlet was cooled by compressed air and the three variable heating zones of the screws were set to values shown in Table 2. The diameter of the extrudates was determined by incident light microscopy.

### 2.5. Microscopy

Light microscopy was performed with a transmission light microscope Zeiss Axiolab (Zeiss, Germany) and the incident light microscope Olympus SZX9 (Olympus, Japan). Photographs were taken by an Olympus UC 30 camera and processed by Stream Motion software.

In order to study the interaction of dry formulations with water, thin layers of the lipid mixtures were spread on slides which were tempered at 37 °C by a heating unit. PBS at 37 °C was added at the edge of the cover glass allowing to follow structural changes of the lipid at the interface solid-liquid by transmitted light microscopy under white and plain polarized light.

### 2.6. Differential Scanning Calorimetry

DSC was performed with a Netzsch DSC 200 (Selb, Germany) with samples from vacuum dried lecithin extrudates. The thermal behavior was determined by heating the extrudates from 10–110 °C with a heating rate of 5 K/min in a closed aluminum pan. The phase transition was defined as the maximum of the second heating curve. Each measurement was run in triplicate.

To obtain information about the thermal behavior of the hydrated extrudates, distilled water was added into the aluminum pan approx. 1:1 (m/m) regarding to the mass of the extrudate. The aluminum pan remained closed to detect the phase transition by heating the samples once from 0−80 °C with 5 K/min. Each measurement was repeated at last once.

Additionally the thermal behavior of drug-loaded lecithin extrudates was determined using a Mettler Toledo DSC 821e (Columbus, OH, USA). To prove the absence of crystalline nicardipine, the appearance of melting peaks was examined during heating from 0–200 °C with 5 K/min in closed aluminum pans.

### 2.7. Texture Analysis/Mechanical Stiffness

Mechanical stiffness of the lecithin mixes was determined by texture analysis (Texture Analyzer CT3-4500, AMETEK Brookfield, Middleboro, MA, USA). Approx. 250 mg of dry lipid mixture were transferred to a cylindrical sample holder (d = 8 mm, h = 5 mm). Measurement was run in single compression mode with a flat cylindrical probe (stainless steel, d = 2 mm, l = 20 mm, m = 5 g) which was inserted into the probe to 3.5 mm depth. Maximum compression force was measured with n = 6 (trigger point 0.005 N, speed 0.1 mm/s) and data was evaluated with TexturePro CT software.

### 2.8. Rheological Measurements

Dynamic oscillatory shear tests were conducted with Kinexus lab+ osciallatory rheometer (Malvern, UK) with a symmetric 20 mm plate-plate geometry and 0.2 mm gap/sample thickness. Dry lipid samples were heated up to 60 °C and spread on the plate, swollen extrudates were measured after 5 days of incubation in PBS at 37 °C and shaking at 50 rpm according to the release setup. The latter were blotted with tissue before spread on the plate. All samples were allowed to settle at 37 °C for 5 min before measurement was started at this constant temperature.

Oscillatory tests were performed to define the elastic and loss moduli, which are calculated as G′ = (τ/γ)*cosδ and G″ = ( τ/γ)*sinδ where τ is the shear stress, γ is the deformation, and δ is the phase shift angle, together with complex viscosity calculated as η* = τ/(γ*ω) where ω is angular frequency. At first, amplitude sweep from 0.01–4% strain was applied at 1 Hz to determine the linear visco-elastic region. Afterwards oscillatory shear measurements as a function of frequency (10–0.1 Hz) were carried out at a constant strain of 0.05%. Data was managed with rSpace.

### 2.9. Quantification of Phosphatidylcholine Types and Nicardipine by ELSD-HPLC

The HPLC system Hitachi LaChrom Elite (Merck, Darmstadt, Germany) is equipped with a HPLC pump L-7100 connected with a quarternary low-pressure gradient mixer and a degasser, the autosampler L-7250 with sample cooler, a column oven L-7360 and a UV detector L-7400. In addition, a low-temperature ELS-detector Sedex 90 LT (Sedere, France) is used to detect all non-volatile compounds with the aim to allow quantification of phospholipids. Data transfer and connection is managed by an interface D-7000 and HSM 700 V 4.1 software.

The mobile phase of the final method consisted of methanol:triethylamine (TEA):acetonitrile 57.6:2.4:40 (*v/v*). TEA is needed to reduce secondary column interactions of the polar PC headgroup and sharpens the peaks [22]. This method was adopted from [23] but developed to the specific needs of the system and the materials in order to reach sufficient separation of the compounds, a high signal to noise ratio and linearity.

30 µL of sample were injected, the flow was set to 1 mL/min and the column temperature was 40 °C. Satisfying separation succeeded with the column LiChroSpher RP-18e LiChroCART 250 × 4 mm 5 µm (Merck, Germany) and a running time of 21 min. Optimized ELSD settings for optimal SNR were: nebulizer temperature 45 °C, gain 4, filter 5 s, nitrogen pressure 3.5 bar. Standard solutions of pure phosphatidylcholine components and nicardipine of known concentrations were made of the compounds in methanol:acetonitrile 60:40 (*v/v*) and analyzed at least in triplicate for gaining a standard curve and calibration.

### 2.10. In Vitro Erosion and Release

Drug loaded and drug free lecithin extrudates were cut into rod-shaped implants of 1 cm length corresponding to a weight of approx. 30 mg. Each implant was inserted in an amber glass vial containing 10 mL of isotonic PBS and these vials were placed into a shaking water bath at 37 °C. For erosion experiments the frequency of shaking was set to 0, 50, 70 and 120 rpm, final release studies were conducted with shaking at 50 rpm.

The following procedure was necessary due to the fragile texture of the implants and the required absence of buffer salts for mass loss determination and analysis by ELSD-HPLC. After predefined time points the implants were taken out carefully and transferred with minimal adhering buffer liquid to tared tubes. The samples were instantly frozen in liquid nitrogen and freeze-dried for 24 h. The tubes with the dried substance were weighed (*m_d_*). To sever the buffer salts, 1 mL of chloroform/methanol 2:1 was added to the solid residue. Preliminary experiments have shown that this mixture is able to solve the lipids and the drug completely but not the interfering salts. After 5 min centrifugation at 13,400 rpm the supernatant containing the analytes was transferred quantitatively to vials and diluted appropriately for HPLC analysis with methanol/acetonitrile 60:40 (*v/v*). The tubes containing the salts were dried for 30 min in vacuum and weighed again (*m_s_*) to calculate the implant’s mass (*m_i_*) after release.

(1) $$m_{i}=m_{d}-m_{s}$$

In consequence, mass loss of the implant due to erosion or dissolution of phospholipids, respectively can be calculated from equation below, based on the initial implant’s mass *m*_0_.

(2) $$mass\;loss\left[\%\right]=\frac{m_{0}-m_{i}}{m_{0}}\times 100$$

Furthermore, changes of the extrudates’ shape were determined by light microscopy.

## 3. Results

### 3.1. Thermal Characterisation of Lipid and Drug-Lipid-Mixtures

**Figure 1:**
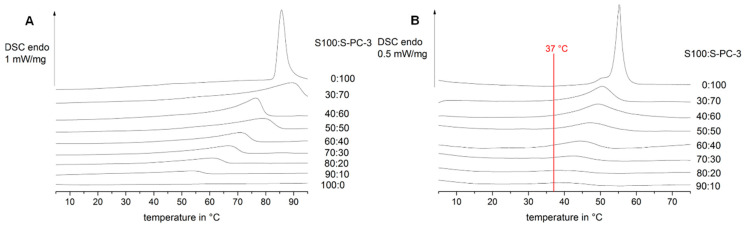
Impact of S100:S-PC-3 ratio on the differential scanning calorimetry (DSC) thermograms in the dry state (**A**) and in the presence of water (**B**).

In a first step, the thermal properties of a variety of mixtures of unsaturated and saturated lecithin were investigated by differential scanning calorimetry (DSC) in the dry state and in the presence of water. Pure S-PC-3 in the dry state has a melting peak at temperatures above 80 °C (Figure 1A top). The presence of water leads to a decrease of the melting peak to temperatures below 60 °C (Figure 1B top). Increasing amounts of the mainly unsaturated phospholipid S100 lead to a broadening and shift of the melting event. Again, melting took place at higher temperatures in dry samples compared to water containing samples. Since lecithin is known as a lyotropic liquid crystalline substance a structural change to bilayer structures in presence of water is likely as well as different phases of the lipids depending on their phase transition temperature and the temperature of the medium.

Since it was the aim of this study to evaluate the suitability of solid pure lecithin mixtures for controlled drug delivery, the phase transition temperature should be above 37 °C to prevent immediate formation of multilamellar vesicles (MLVs). For this reason, the mixtures in a range from 40:60–60:40 S100:S-PC-3 were selected for further studies. Mixtures with a S100 content above 60% have low melting temperatures which impact the extrudate characteristics (they become too soft and sticky) and would lose their geometry after implantation. Mixtures with a S100 content below 40% were excluded from further investigations in this study, because they showed in preliminary studies very low water uptake and erosion, which is expected to be too slow for the desired nicardipine release period of 3–4 weeks. They might be interesting systems for extended release periods (months) for other indications.

The interaction of the phospholipid with water should lead to water uptake in the phospholipid matrix and formation of vesicles at the phospholipid-buffer interface. As mentioned above this formation is assumed to be dependent from the composition of the sample and the medium temperature which was tested in preliminary studies at 25 °C, 37 °C and 50 °C since vesicle formation is only possible above the lipids’ phase transition temperature. At 37 °C all the mentioned lipid mixtures for further investigation are showing thermal events according to Figure 1 and vesicle formation could be possible. To prove or disprove this assumption, the behavior at the extrudates’ surface was followed with light microscopy at 37 °C (body temperature).

**Figure 2:**
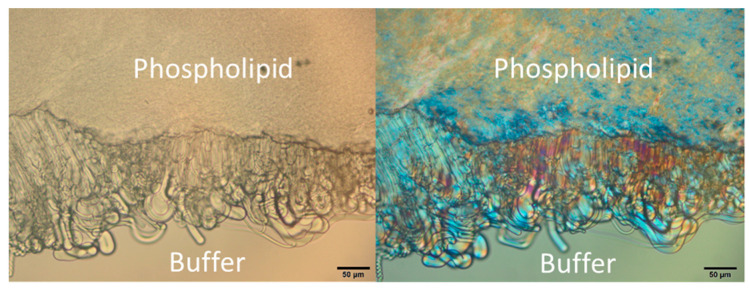
Formation of vesicles from S100:S-PC-3 50:50 at 37 °C at the interface of lipid mixture and buffer in white light transmission mode (**left**) and polarization mode (**right**). The black scale bar represents 50 µm.

As an example, Figure 2 shows the S100:S-PC-3 50:50 mixture shortly after addition of buffer to the edge of the sample. Vesicle-like structures emerge at the phospholipid/buffer interface. They show also birefringence and maltese crosses which are typical for lamellar phases. All analyzed mixtures exhibited this behavior of vesicle formation in presence of buffer but formation was more pronounced with a higher share of S100. Movement of the sample lead to detachment of the superficial lamellar structures resulting in liposomes in the medium and with regard to the extrudates by that in material and mass loss and a possible way of drug release. In consequence, the impact of shear stress needs to be evaluated since the budding off and pinching out of the vesicles is triggered by shear stress and depending on the implantation site. The results of investigations of the mechanical properties are presented in Section 3.3.

**Figure 3:**
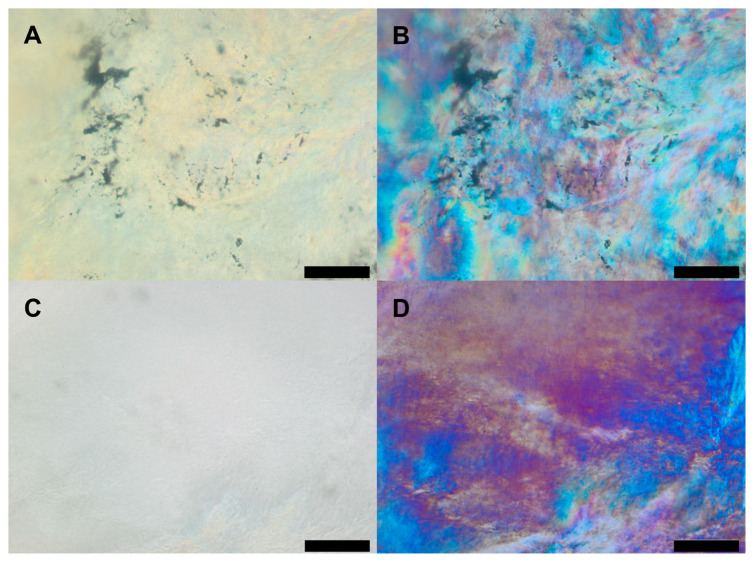
Transmitted light microscopy images without (**A**,**C**) and with (**B**,**D**) polarized light of lecithin mixtures S100:S-PC-3 50:50 containing 10 wt % nicardipine (**A**,**B**) or nicardipine hydrochloride (**C**,**D**) respectively. The black scale bars represent 100 µm.

In a next step, the impact of drug incorporation was studied. Nicardipine was incorporated either in the form of the drug base or in the form of the hydrochloride. Light microscopic pictures of the 50:50 mixture containing 10% API indicate the absence of drug crystals (Figure 3) and are shown exemplarily for all further investigated mixtures from 40:60 to 60:40 S100:S-PC-3. Microscopic pictures obtained with polarized light indicate the presence of an anisotropic birefringent material (Figure 3B,D) which could be attributed to lamellar or hexagonal liquid crystalline phases. The whole examined dry samples exhibited a homogeneous appearance with no further ordered structure detectable by polarized light microscopy. The same structural pattern was observed for pure S100 and S-PC-3 and mixtures thereof (data not shown).

**Figure 4:**
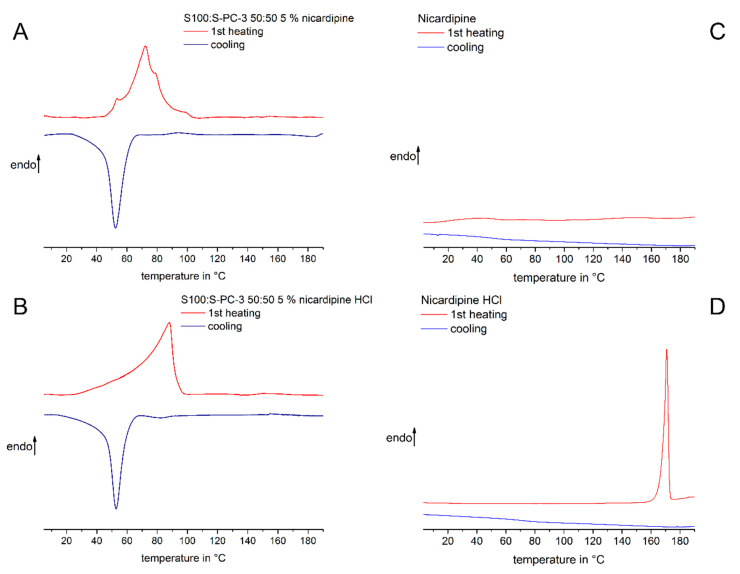
DSC thermograms of lecithin mixture S100:S-PC-3 50:50 containing nicardipine free base (**A**) or nicardipine hydrochloride, respectively (**B**) show no melting events of the drug in the phospholipid system. DSC measurements of pure nicardipine hydrochloride (**D**) lead to a distinct melting peak and formation of a supercooled melt after cooling. On contrary, DSC analysis of nicardipine free base (**C**) shows no thermal events in the recorded temperature range.

The thermal properties of nicardipine base, nicardipine hydrochloride and the drug loaded phospholipid mixtures were investigated by DSC (Figure 4). Nicardipine hydrochloride showed a melting peak around 170 degrees Celsius (Figure 4D), which agrees with values from the literature [24] and chemical suppliers. No recrystallization occurred during the cooling, which indicates the high potential of the drug to form supercooled melts. Nicardipine base did not show any thermotropic event (Figure 4C), which was already experienced in our previous study [6] as well as by other groups [25]. Drug loaded phospholipid samples did show melting and recrystallization events (Figure 4A,B). For the nicardipine hydrochloride loaded sample, melting was observed in the temperature range between 20 and 100 °C. For the nicardipine base loaded sample, melting occurred between 50 and 110 °C. The cooling curves for both samples were quite similar and indicated recrystallization of the phospholipid drug mixtures. No thermal events in the temperature range of the pure drug were observed. Therefore, the DSC studies support the findings of the light microscopic investigations and indicate that both drug molecules are not just suspended as drug crystals in the phospholipid matrix, but exist in a non-crystalline, homogeneous distribution within the liquid crystalline phospholipid matrix.

### 3.2. Determination of Sample Composition by HPLC-ELSD

**Figure 5:**
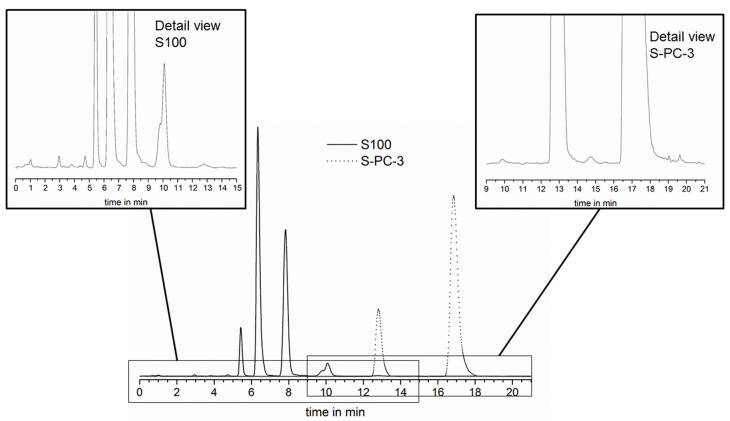
HPLC elution profile of Lipoid S100 and Lipoid S-PC-3 with the developed method in overlay and detail view of the less pronounced peaks.

In a next step, a HPLC method was established in order to monitor the phospholipid composition and the drug concentration. The detection of single phospholipid components would enable the monitoring of the phospholipid composition during the release. By this means it would be possible to answer the question, if the phospholipids erode simultaneously or if single components are preferentially released. The evaporative light scattering detector (ELSD) does not require substances with UV- or VIS-absorption and enables the detection of components which are otherwise difficult to monitor (e.g., lipids and phospholipids) [26,27]. With the applied method, phospholipids with unsaturated fatty acids elute faster compared to the saturated counterparts and could be separated by their equivalent carbon number [23]. Using chemical pure standards, the assignment of the phospholipid components was possible for S100 and S-PC-3 (for details, see Supplement Figure S1), the main components of the saturated phospholipid S-PC-3 were identified as 1,2-distearoyl-sn-glycero-3-phosphocholine (18:0–18:0 PC, DSPC) and 1-palmitoyl-2-stearoyl-sn-glycero-3-phosphocholine (16:0–18:0 PC, PSPC); 1,2-dilinoleoyl-sn-glycero-3-phosphocholine (18:2–18:2 PC, DLPC) and 1-palmitoyl-2-linoleoyl-sn-glycero-3-phosphocholine (16:0–18:2 PC, PLPC) were chosen as reference compounds to describe composition of S100 well. For S100 and S-PC-3 mixtures, the HPLC method separated the peaks of all four chosen model compounds very clearly (Figure 5) and calibration curves with the chemical pure standards could be recorded (see supplement Figure S2) showing a non-linear correlation as described in literature for the applied amount of substance [26].

**Figure 6:**
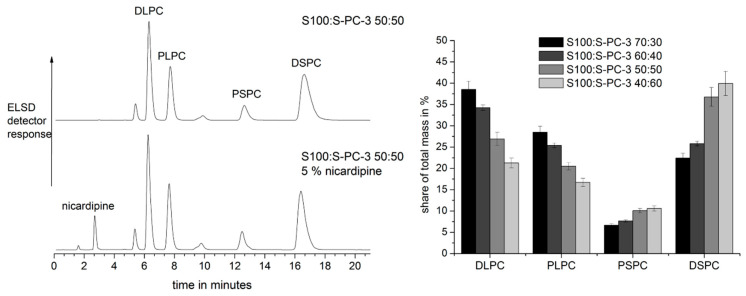
HPLC chromatograms of drug free (top) and drug loaded (bottom) S100:S-PC-3 50:50 mixtures (**left**) and analysis of the composition of S100:S-PC-3 extrudates with different lecithin type shares (**right**; mean ± SD, *n* = 3).

It was therefore possible to quantify single components for different phospholipid mixtures (Figure 6 left top). Decreasing contents of S100 led to a decrease of DLPC and PLPC and to an increase of PSPC and DSPC (Figure 6 right). Furthermore, the nicardipine peak was well separated from the phospholipid peaks in the HPLC chromatogram. Therefore, in addition to the phospholipid composition, the drug concentration could be determined in the same run (Figure 6 left bottom) and was detectable by the ELS- and UV-detectors. In the next step, the phospholipid samples were exposed to isotonic phosphate buffer and erosion and release studies were conducted.

With the help of this quantitative analysis also homogenous drug distribution within the lipid mixtures could be verified resulting in nicardipine contents of 5.16–5.77% and relative standard deviations from measurement of 6 different extrudates per batch from 0.86% to 2.54%.

### 3.3. Mechanical Characterization of the Samples and Erosion Studies

**Figure 7:**
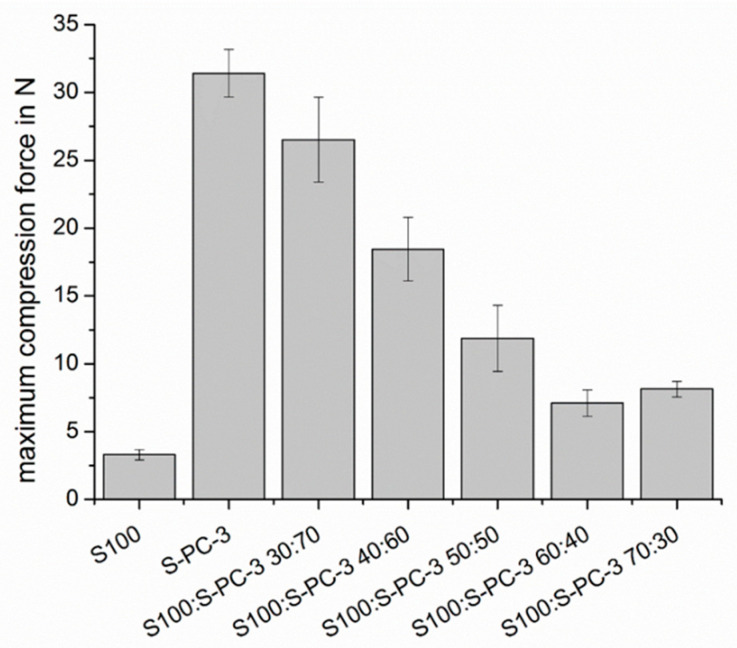
Maximum compression force during insertion of a cylindrical tool (d = 8 mm) into different lecithin mixtures as a parameter for their mechanical stiffness.

In a first step of the investigations of the mechanical properties, different ratios between the S100 and S-PC-3 were investigated by texture analysis in order to evaluate mechanical stability thereof. The maximum compression force for an 8 mm cylinder was slightly above 30 N for pure S-PC-3 (see Figure 7). For pure S100, which is rich in unsaturated fatty acids, much smaller values (<4 N) were observed. The compression forces of the phospholipid mixtures were between these two values. The sample with higher S-PC-3 (Figure 7). content showed still compression forces above 25 N. However, values below 10 N were observed for mixtures with a content of S-PC-3 content below 40% which caused less facile handling of the resulting implants.

Mechanical stress in vivo is expected to be dependent from the implantation site. It will be low for brain implants and larger for subcutaneous implants. For this reason, oscillatory rheological measurements were performed either with dry or buffer exposed samples of the lipid mixtures from 60:40 to 40:60 S100:S-PC-3 and also with 5% (*w/w*) nicardipine containing samples of S100:S-PC-3 50:50.

**Figure 8:**
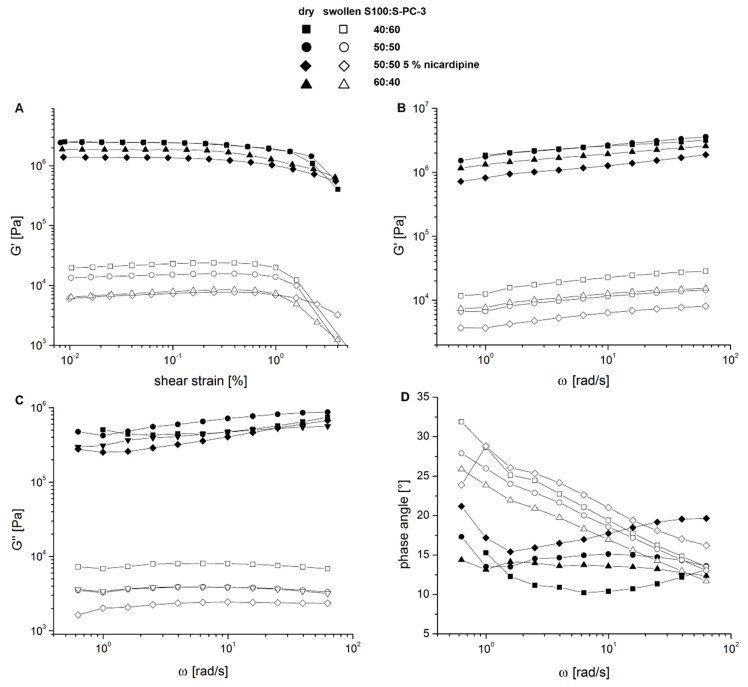
Results of rheological measurements of different S100-S-PC-3 mixtures either in dry or swollen state after 5 days of incubation in PBS at 37 °C and 50 rpm shaking. The course of the storage modulus G′ during amplitude sweep showed a linear visco-elastic behavior until approx. 1% strain (**A**). During frequency sweep the course of storage modulus G′ (**B**), loss modulus G″ (**C**) and phase angle δ (**D**) could be followed showing differences of the rheological properties of the samples in dry and swollen state.

In a first step, an amplitude sweep was applied to the samples. Figure 8A shows that the elastic modulus (G′) remains constant until a shear strain of 1% both for the formulation in dry and wet state. This range indicates the ideal visco-elastic range. In case of the wet samples a slight increase in G′ is visible which might be attributed to protrusion of water out of the specimen due to shear stress. By application of shear strain higher than 1% both storage as well as loss modulus (data not shown) decrease significantly. The decrease of the moduli during amplitude sweep is more pronounced for the swollen samples. Phase angles <22° were detected indicating a higher elastic share and gel-like behavior. Supplementary Figure S3 provides an example of the course of G′, G″ and δ during amplitude sweep measurements.

For investigations in the frequency sweep mode a shear strain of 0.05% was applied and results in a slight increase of the storage modulus while increasing the shear frequency (Figure 8B) and also in an increase of the loss modulus in case of the dry samples (Figure 8C) whereas for the swollen samples nearly constant loss moduli were detected. Storage moduli ranged from 700–3000 kPa for the dry samples and 2–20 kPa for swollen samples, the complex viscosities differ in the same magnitude.

The drug containing sample behaves differently compared to the S100:S-PC-3 50:50 mixture without drug during the rheological measurements. Since within the dry samples the 40:60 and 50:50 samples show nearly the same behavior the 60:40–formulation exhibits a slightly lower storage modulus and complex viscosity. However, the 50:50 sample with nicardipine exhibits an overall reduced viscosity compared to the other samples which comes more evident in the swollen state and is likely due to the replacement of viscous lipid with drug. In this state after incubation in water, the 50:50 sample without drug also shows not a behavior comparable to the 40:60 mixture anymore but behaves more like the 60:40 sample with the higher share of unsaturated lipids.

Having a look on the phase shift angles δ during frequency sweep testing (Figure 8D) a further difference between the dry and swollen samples becomes evident unless the fact that all samples are more elastic than viscous. Whereas in case of the dry samples the loss modulus also is slightly increasing with higher shear frequency the resulting phase angles are slightly altering without a clear trend between 10–22° indicating a more elastic system with a high viscosity. A slight initial decrease of δ at 0.6-2 rad/s was observed. In case of the swollen samples for all formulations an increased δ is visible with lower oscillation frequency indicating a more viscous behavior at lower frequencies and a more elastic system at higher shear frequency.

**Figure 9:**
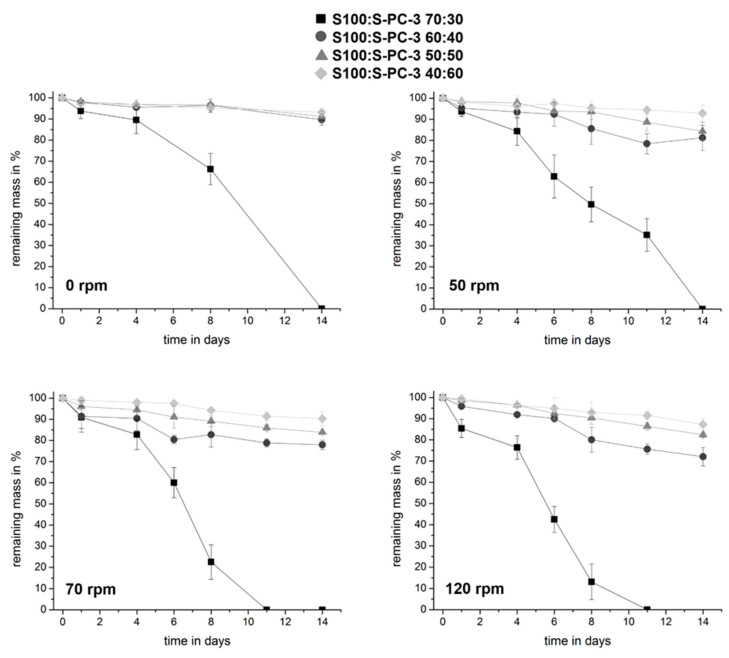
Impact of composition and shear stress on time dependent erosion after exposure to buffer. Data are presented as mean ± SD, *n* = 4.

Depending on the implantation site, implants may be exposed to quite different degrees of mechanical stress. For many drug delivery systems, mechanical stress may have a large impact on the erosion of the implant and the release rate. In order to identify the impact of the differences of the mixtures detected by rheology on the erosion behavior we studied the impact of different shearing rates on the erosion of different phospholipid implants in vitro (results shown in Figure 9).

**Figure 10:**
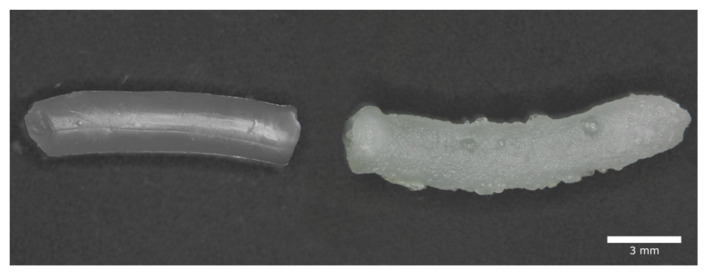
Influence of shear stress on the transformation of extrudates during incubation. Shown are two extrudates with a ratio of Lipoid S100 and Lipoid S-PC-3 of 50:50 after 4 days of incubation. The right extrudate was exposed to no shear stress and the left one was exposed to shaking with 50 rpm.

The S100:S-PC-3 70:30 implant did erode even without any mechanical stress within 14 days. Increasing shear stress accelerated the process. For this reason, this formulation was excluded from drug release studies. All other implants had a higher stability and kept over 80% of their mass after two weeks. They behaved very similar under stress free release conditions. Differences became visible at higher shear rates. As expected, higher contents of the solid phospholipid S-PC-3 led to decreased erosion. After exposure to buffer, implants change slightly their form due to swelling (Figure 10). The sample that has not been exposed to any shear stress (Figure 10 right) shows a rough surface with adhering bubble-like structures. On contrast, the sample incubated in the same medium with shaking at 50 rpm shows a smooth shiny surface.

**Figure 11:**
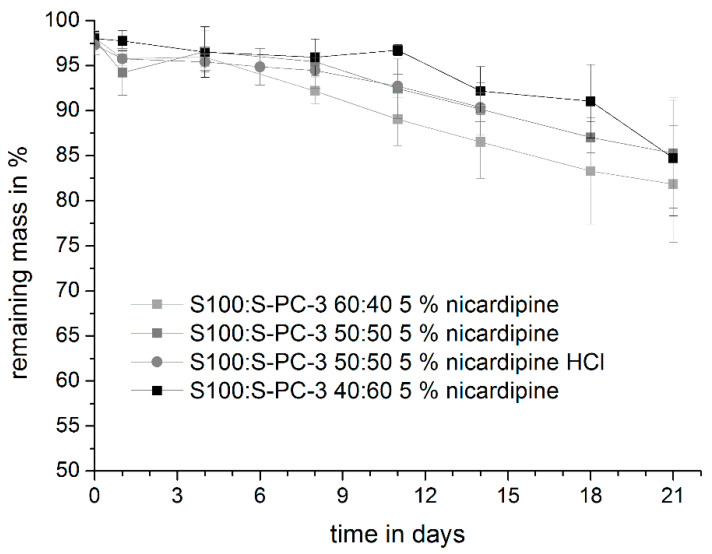
Mass loss profile of S100:S-PC-3 extrudates containing 5% nicardipine (HCl) during the release study in PBS pH 7.4 at 50 rpm. Data are presented as mean ± SD, *n* = 3.

The presence of the drug nicardipine base or nicardipine hydrochloride (drug load 5%) did not change the erosion pattern of the phospholipid extrudates (Figure 11). Similar to drug-free systems, extrudates with a higher amount of S100 eroded faster.

### 3.4. Drug Release Studies

**Figure 12:**
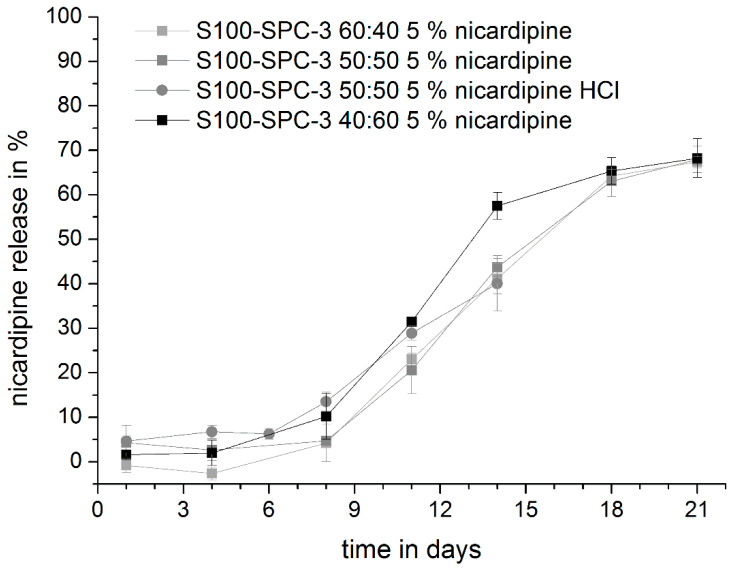
Release of nicardipine from S100:S-PC-3 extrudates of different composition. Release was studied at 37 °C, isotonic PBS pH 7.4 and 50 rpm. Data are presented as mean ± SD, *n* = 3.

In the next step, release studies were conducted. Very similar release profiles were observed for nicardipine loaded extrudates 60:40, 50:50 and 40:60. No striking difference were observed between the 50:50 implants loaded with nicardipine base or hydrochloride (Figure 12). After a lag time of about five days, nicardipine was released continuously in the following two weeks. Therefore, the release profile shows a good match with the desired profile for the prevention of vasospasms.

**Figure 13:**
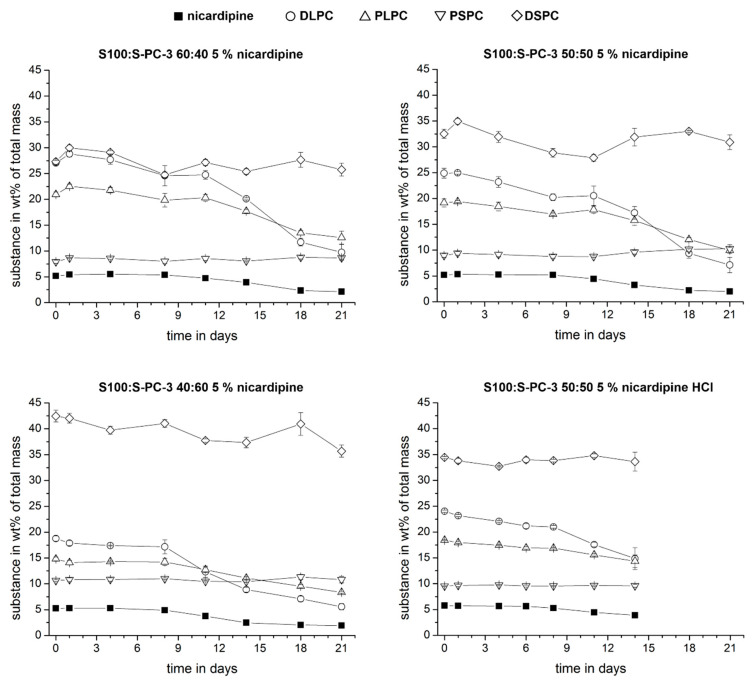
Relative fraction (*w/w*) of drug and model compounds in lecithin extrudates during release studies at 50 rpm and 37 °C in PBS. Data are presented as mean ± SD, *n* = 3.

In order to get a deeper insight into the release mechanisms, we used the established HPLC method to monitor the amount of single phospholipid components in the remaining extrudates to compare it with the remaining drug. If pure erosion would control the release, all phospholipid components and the drug would be released at the same rate. The percentage of each component in the remaining extrudate would be constant over time. A diffusion controlled release of a single or several species would result in a decrease of their content in the remaining mass. The time dependent percentages of the individual phospholipid components and the drug in the remaining mass are shown in Figure 13. For all samples, the drug content decreased over time which indicates that diffusion processes contribute, at least partially, to the release mechanism. For the phospholipid components, only the DLPC (1,2-dilinoleoyl-sn-glycero-3-phosphocholine) showed a remarkable drop in all samples. Due to its chemical structure with two doubled unsaturated fatty acids (18:2–18:2 PC) this molecule will have a higher mobility compared to the other phospholipid components. The kinetic profile of DLPC and nicardipine are quite similar. With time, we observed that the peak area of the standard peaks decreased and the area of the additional peaks (degradation products) increased (Supplement Figure S4). This is probable caused by hydrolysis and or oxidation processes.

## 4. Discussion

We explored the possibility to use phospholipid extrudates for parenteral controlled release. In the first step, different mixtures between the phospholipid S100 (semi-solid/plastic solid) and S-PC-3 (solid) were produced and investigated for their potential as preformed implant. The results of the DSC studies indicate the strong impact of the S100:S-PC-3-ratio and presence or absence of water on the thermal transitions. A minimum of 40% of the solid and saturated phospholipid S-PC-3 is required to achieve sufficient mechanical stability for processing and handling and to prevent a melting of the extrudate at body temperature. The lipophilic drug nicardipine was embedded in a non-crystalline state in the lipid mixtures since no corresponding melting peak in DSC and no presence of drug crystals in polarized light microscopy was visible as this was the case for nifedipine in preliminary studies. Light microscopy showed the presence of lyotropic phases at the phospholipid-buffer interface.

Results of texture analysis, DSC and erosion studies in buffer indicated, that a S-PC-3 content of 50% and above is required for a certain mechanical stability of the implants but will result in a thermal phase transition of the mixture above 37 °C. We incorporated nicardipine base or nicardipine hydrochloride at 5% successfully. A release of several weeks was achieved in vitro. A lag time of about five days was observed. In most cases, the vasospasms do not occur during the first days in subarachnoid hemorrhage. We do also expect, that the release profile might be slightly faster in vivo due to the presence of enzymes and a better solubility of the phospholipids in the surrounding matrix, furthermore the corresponding mechanical stress in vivo needs to be taken into account. The lag time could also be shortened by the addition of excipients, which will increase the initial water uptake. The present study shows clearly the general feasibility of phospholipid implants for parenteral controlled release and the release and erosion kinetics can be tuned in both directions.

In our previous studies of nicardipine release form PLGA implants we observed a faster erosion of PLGA compared to nicardipine (and also nifedipine) release, which indicated that the drug release was controlled by the poor solubility of the drug, but not by the polymer matrix [6]. With the phospholipid extrudates, a different behavior was observed. Using HPLC with an ELSD detector, we were able to measure the drug content and the percentage of specific phospholipids. In contrast to PLGA, the drug content (%) decreased with time. A similar pattern was observed for the unsaturated phospholipid DLPC. We therefore conclude that drug release occurs after water uptake (=lag time), which leads to formation of laminar phases and the following diffusion of DLPC with the solubilized nicardipine through the partially solid phospholipid network. Compared to PLGA, a better release profile was achieved. The avoidance of the formation of hydrophilic and highly acidic degradation products is an important advantage of phospholipids compared to PLA/PLGA systems. It is expected that this feature will be beneficial for parenteral controlled release formulations of peptides and other acid labile drugs. With respect to the release of poorly water soluble drugs, it might be an advantage that possible degradation products of the phospholipids (e.g., fatty acids and lysolecithin) are amphiphilic and therefore potential solubilizers for these molecules. In contrast, lactic and glycolic acid, the PLGA degradation products are highly hydrophilic molecules. Within this study, the delivery matrix was composed of two different phospholipid excipients, the mainly unsaturated S100 and the saturated S-PC-3. An amount of >40% of S-PC-3 was necessary to ensure sufficient mechanical stability and a solid character at body temperature. S100 aids the water uptake and formation of vesicles at the phospholipid/buffer interface and leads to a faster erosion. Phospholipid implants with a high S-PC-3 and low S100 content are interesting candidates for prolonged release times. Enzymatic degradation might play an important role for the in vivo fate of the extrudates. Therefore, future studies will be devoted to the monitoring of the biofate of the developed extrudates and the release kinetics of drugs and model drugs to get information about the similarity or discrepancy between the in vitro and in vivo situation.

## 5. Conclusions

In conclusion, we propose phospholipid implants are an attractive option for parenteral controlled drug delivery. The high biocompatibility and safety make phospholipids very attractive as PLA and PLGA alternatives. In addition, phospholipid hydrolysis will not lead to highly acidic degradation products which cause autocatalytic degradation processes and also acylation or degradation of drugs prior release. The current study demonstrates the general feasibility in vitro and shows for nicardipine loaded extrudates advantages compared to previously investigated PLGA systems [6]. However, further in vitro and also in vivo studies are required to explore the potential of phospholipid extrudates and to get more information about the in vitro/in vivo correlation of release mechanisms and kinetics.

## Figures and Tables

**Table 1:** Typical fatty acid composition of natural soybean lecithin (provided by manufacturer Lipoid).

Fatty Acid	Unsaturated Lecithin Lipoid S100	Hydrogenated Lecithin Lipoid S-PC-3
16:0 (palmitic)	15%	13%
18:0 (stearic)	3%	86%
18:1 (oleic and isomers)	12%	<1%
18:2 (linoleic)	62%	
18:3 (linolenic)	5%	

**Table 2:** Extrusion parameters for manufacturing of lecithin extrudates.

Mixture Composition S100:S-PC-3	Extrusion Speed in Rpm	Temperature Setting of the Three Heating Zones in °C
70:30	100	26/26/28
60:40	100	28/28/30
50:50	100	32/32/36
40:60	100	34/34/36

## Supplementary Materials

The following are available online at https://www.mdpi.com/1999-4923/12/9/817/s1, Figure S1: HPLC chromatograms of (A) S100 and (B) S-PC-3 each with selected standard PC types, Figure S2. ELSD-HPLC calibration curves of phosphatidylcholine standards DLPC (A), PLPC (B), PSPC (C) and DSPC (D) as well as nicardipine hydrochloride (E), Figure S3. Impact of shear strain and buffer exposure (5 days of incubation in PBS at37 °C) on the phase angle and the elastic (G′) and loss (G″) moduli of S100:S-PC-3 60:40, Figure S4. Percentage of identified species from total extrudate mass during release studies. Data are presented as mean ± SD, n = 3.
