# Supplementary material for: Nicardipine Loaded Solid Phospholipid Extrudates for the Prevention of Cerebral Vasospasms: In Vitro Characterization
Source: Pharmaceutics. 2020 Aug 28;12(9):817. doi: 10.3390/pharmaceutics12090817 (PMC7557531; doi:10.3390/pharmaceutics12090817)
Supplement: Supplementary file 1 [file pharmaceutics-12-00817-s001.pdf]

Supplementary Materials

Nicardipine Loaded Solid Phospholipid Extrudates for the Prevention of Cerebral Vasospasms: In Vitro Characterization

Christin Zlomke, Johannes Albrecht and Karsten Mäder

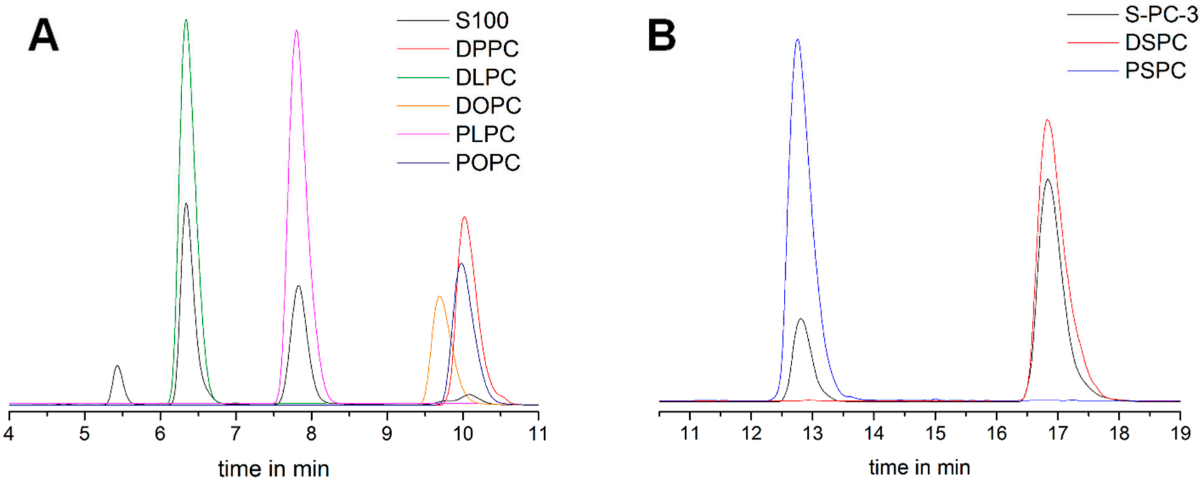

Figure S1. HPLC chromatograms of (**A**) S100 and (**B**) S-PC-3 each with selected standard PC types.

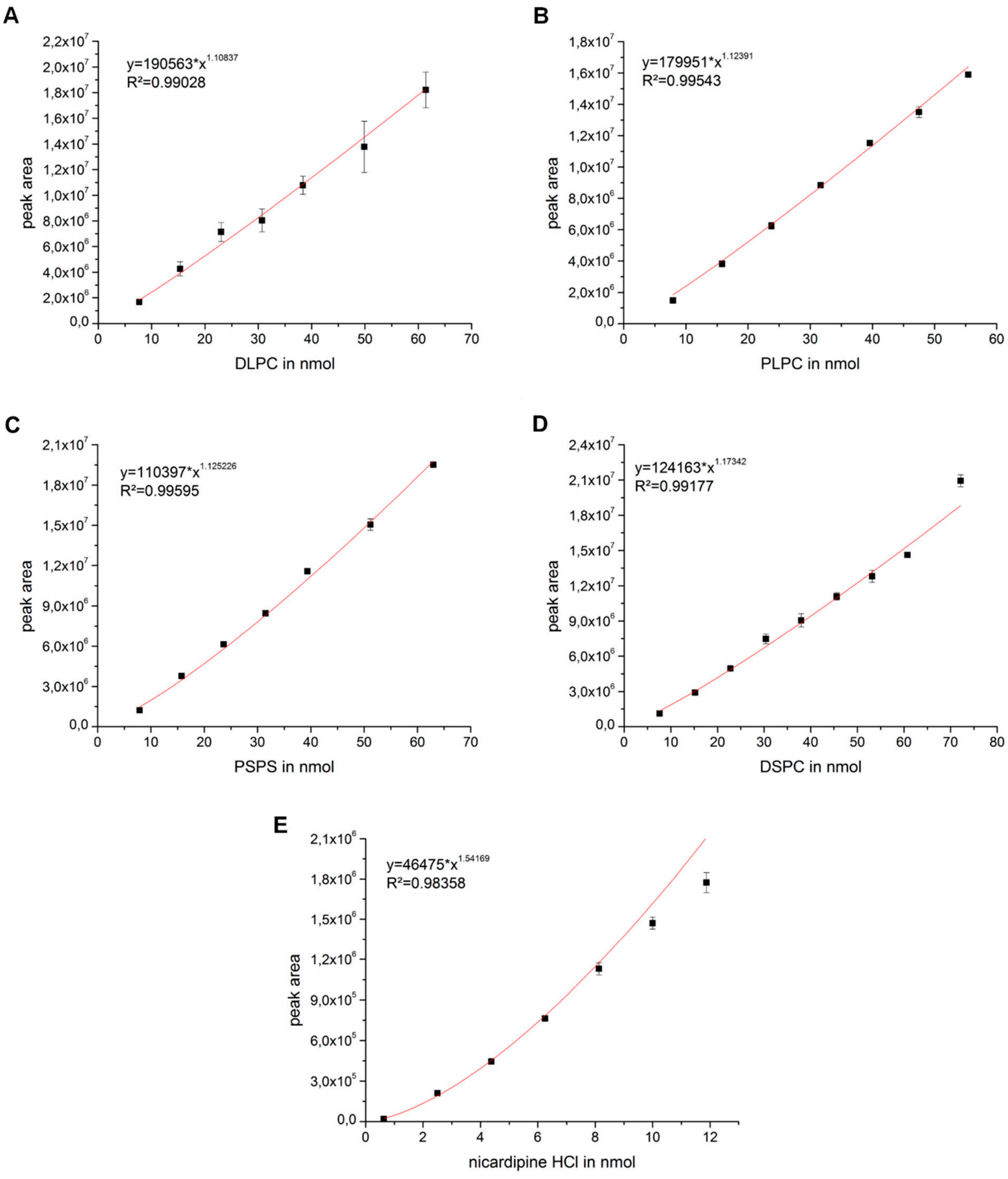

Figure S1. ELSD-HPLC calibration curves of phosphatidylcholine standards DLPC (**A**), PLPC (**B**), PSPC (**C**) and DSPC (**D**) as well as nicardipine hydrochloride (**E**).

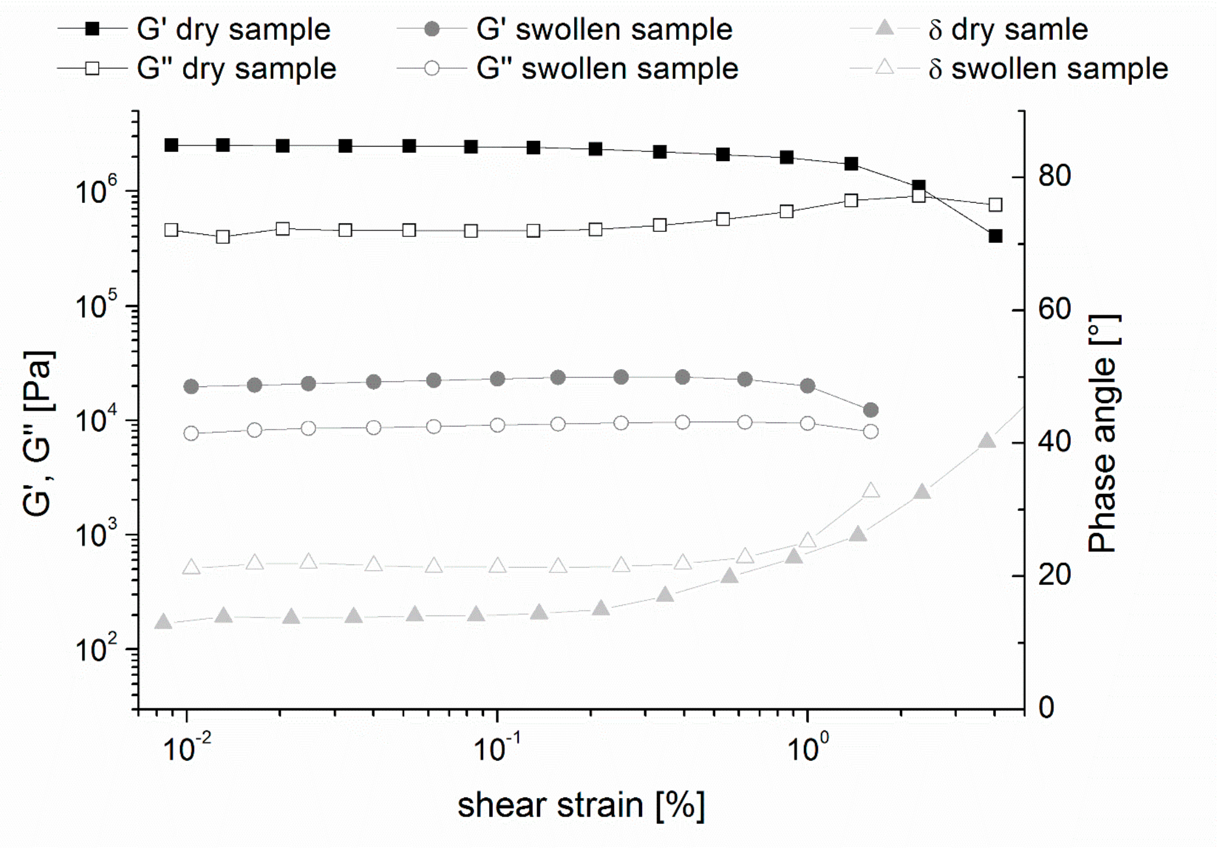

Figure S2. Impact of shear strain and buffer exposure (5 days of incubation in PBS at37 °C) on the phase angle and the elastic (G′) and loss (G′′) moduli of S100:S-PC-3 60:40.

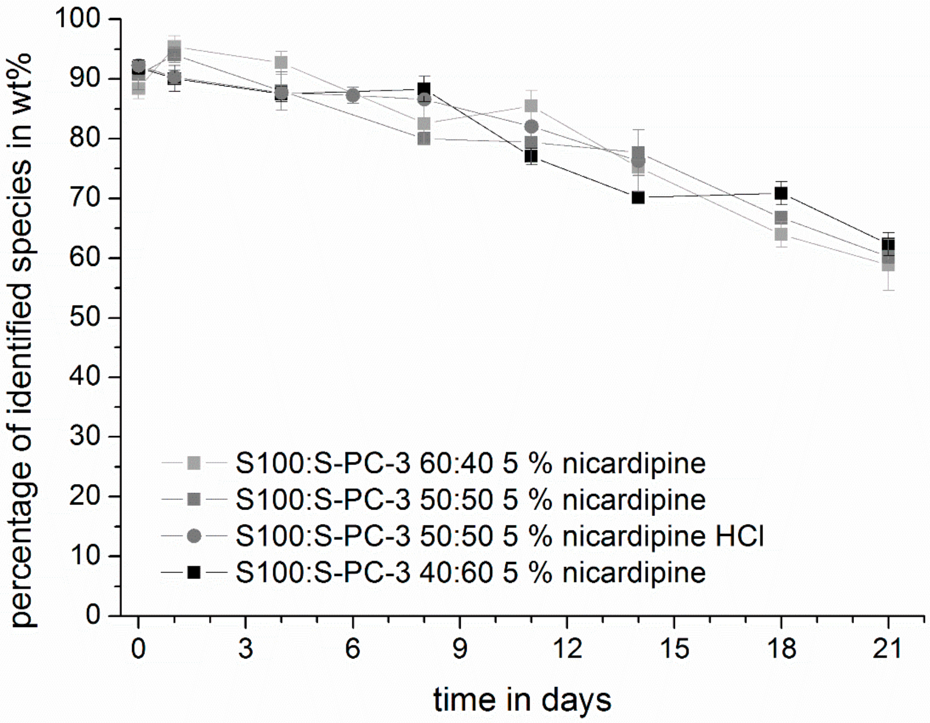

Figure S4. Percentage of identified species from total extrudate mass during release studies. Data are presented as mean ± SD, *n* = 3.
